# Women with HIV in Indonesia: are they bridging a concentrated epidemic to the wider community?

**DOI:** 10.1186/s13104-015-1748-x

**Published:** 2015-12-09

**Authors:** Annisa Rahmalia, Rudi Wisaksana, Hinta Meijerink, Agnes R. Indrati, Bachti Alisjahbana, Nel Roeleveld, Andre J. A. M. van der Ven, Marie Laga, Reinout van Crevel

**Affiliations:** Tuberculosis and HIV Research Centre, Faculty of Medicine Universitas Padjadjaran, Jl. Dr. Eijkman No. 38, Bandung, 40161 Indonesia; Department of Internal Medicine, Radboud University Medical Centre, Nijmegen, The Netherlands; Department of Internal Medicine, Faculty of Medicine Universitas Padjadjaran, Bandung, Indonesia; Department of Clinical Pathology, Faculty of Medicine Universitas Padjadjaran, Bandung, Indonesia; Department for Health Evidence, Radboud University Medical Centre, Nijmegen, The Netherlands; Department of Paediatrics, Radboud University Medical Centre, Nijmegen, The Netherlands; Department of Public Health, Institute of Tropical Medicine, Antwerp, Belgium

**Keywords:** HIV, Women, Reproductive health, Concentrated epidemic, Indonesia

## Abstract

**Background:**

Male injecting drug users drove the onset of the HIV epidemic in Indonesia but over time more women have been diagnosed. We examined the relative proportion of female patients in an HIV cohort and characterized their probable transmission route and reproductive profile.

**Designs:**

Prospective cohort study in a referral hospital in West Java.

**Methods:**

Interviews with standardized questionnaires, physical and laboratory examinations were done for 2622 individuals enrolled in HIV care between 2007 and 2012. The proportion of women in this cohort was compared with national estimates. The general characteristics of HIV-infected women and men as well as the sexual and reproductive health of HIV-infected women were described.

**Results:**

The proportion of female patients enrolled in HIV care increased from 22.2 % in 2007 to 38.3 % in 2012, in line with national estimates. Women were younger than men, fewer reported a history of IDU (16.1 vs. 73.8 %, *p* < 0.001) and more were tested for HIV because of a positive partner (25.5 vs. 4.0 %, *p* < 0.001). The majority of women were in their reproductive age, had children, and were not using contraceptives at the time of enrolment.

**Conclusion:**

HIV-infected women in Indonesia have specific characteristics that differ them from women in the general population. Further research to elucidate the characteristics of women exposed to HIV, their access to testing and care and sexual and reproductive needs can help reduce transmission to women and children in the context of concentrated HIV epidemic in Indonesia.

## Background

Indonesia has a relatively recent but rapidly growing HIV epidemic that, apart from the Papua provinces, is concentrated in some key populations. According to the Indonesia AIDS Commission the number of HIV-infected individuals increased at least three-fold between 2009 and 2014 [1]. An integrated biological and behavioural survey on key populations in 2011 found 41 % HIV prevalence among people who inject drugs (PWID), 10 % among direct female sex workers (FSW) and 8 % among men who have sex with men (MSM) [2]; UNAIDS in 2012 estimated a lower prevalence among PWID (36.4 %) and FSW (7 %) and a similar estimate in MSM (8.5 %) [3]. By 2014, the male-to-female ratio of cumulative AIDS cases in Indonesia was 1.8:1 [4].

Several studies have predicted the transition from a concentrated to a more generalized HIV epidemic due to the sexual behaviour of key populations [5–7], as has been observed in other settings [8]. In Indonesia, new HIV cases are projected to increase at a higher rate in men than women due to HIV incidence among MSM [9]. However, female partners of men who inject drugs [10] and MSM [6] may not be considered at risk for HIV and remain undiagnosed until they develop symptoms or lose a child due to HIV/AIDS. The delay in HIV detection in women poses a risk of not addressing the sexual and reproductive health (SRH) issues [11–13] important to prevent further transmission to children or other partner(s).

The prevention of mother-to-child transmission (PMTCT) program in Indonesia does not include HIV screening at antenatal care [14]. There is little information on seropositivity among pregnant women in the general population. Previous studies reported a 0 % HIV cases among 2450 pregnant women in Bali [15], 0.41 % among 11,693 screened in eight cities between 2003 and 2010 [16] and 2.5 % among 21,103 tested according to 2011 national report [1].

Investigating the transmission route of women infected with HIV and their risk of transmitting HIV to their children and/or sexual partners can help make epidemiological predictions and identify health needs of HIV-infected women in Indonesia. We therefore examined the ratio of men and women in a cohort of HIV patients [17] in a teaching hospital over a five-year period, and characterized the probable transmission route and reproductive profile of HIV-infected women in this cohort.

## Methods

### Setting and study population

The study was performed at Hasan Sadikin hospital, the main referral hospital in Bandung, the capital of West Java, a province of 43 million [18] in Indonesia, which has one of the highest HIV rates in Indonesia. Since August 2007 all subjects enrolled in HIV care have been included in a prospective cohort [19]. The Health Research Ethics Committee of the Faculty of Medicine Padjadjaran University in Bandung, Indonesia approved the study, and all patients provided written informed consent. As one of the first 25 hospitals selected by the government to provide HIV care, this hospital has delivered free antiretroviral therapy (ART) since December 2004. CD4 testing became available in Hasan Sadikin hospital in September 2007 while measurement of HIV-RNA can be done since January 2008.

HIV testing was done through voluntary counselling and testing (VCT) or, when patients were referred from other departments within the hospital, through provider-initiated testing and counselling (PITC). HIV-infected patients were enrolled in care and given cART according to the national guidelines. Women who were found pregnant when entering HIV care were given prophylactic ARV and referred to the obstetrics department. HIV screening in antenatal clinics is no routine in West Java, including in the main referral hospital.

### Clinical procedures

Data on demographic factors and probable HIV transmission route were collected through interviews with standardized questionnaires. Questions about risk behaviour included inquiries about injecting drug use but not about commercial sex work—information about sex work was obtained only if patients mentioned ‘clients’ as one of their sexual partners. The attending physician examined patients to assess their clinical condition and possible co-morbidities but pregnancy tests were not routinely done.

### Laboratory procedures

Blood samples were taken for serological testing on HIV, hepatitis B virus (HBV), hepatitis C virus (HCV) and measurement of the CD4 cell-count per ml blood. HIV antibodies were measured using commercially available rapid tests (Determine HIV-1/2, Abbott laboratories, Tokyo, Japan; SD HIV-1/2 3.0, Standard Diagnostic, Inc, Kyonggi-do, Korea); enzyme immunoassay (EIA; Virolisa, Index Union Diagnostic, Korea); and electrochemiluminescence immunoassay (ECLIA; HIV combi, Roche, Mannheim, Germany) in accordance with national guidelines. HBsAg, anti-HBs, anti-HBc and anti-HCV were measured by ECLIA (Roche diagnostic, Mannheim, Germany). External quality control of HIV, HBV and HCV serology (National Serology Reference Laboratory, Australia) showed 100 % accuracy. CD4 cell measurements were taken using Facscount flow cytometry technology (BD Biosciences, Jakarta, Indonesia). External quality assurance for CD4 measurement was performed from COE Thailand and Qasi Canada (SD < 1 %).

### Statistical analyses

Each patient was assigned a code and all data were collected on standardized forms using the patient code and subsequently entered in a central database in Microsoft Access. A total of 2833 individuals above 16 years of age—who were not recruited from the main narcotic prison of West Java [20]—had been recorded on March 31, 2013. This study analysed individuals enrolled in the HIV cohort between August 1, 2007 and December 31, 2012. Ten individuals (0.4 %) who were already in HIV care before the cohort was started, 94 (3.3 %) who entered the cohort after 2012, and 67 (2.4 %) whose baseline variables were missing were excluded. Finally, 2662 individuals, i.e. 1781 (67 %) men and 881 (33 %) women were included for further analysis.

The proportion of women enrolled in HIV care in this hospital was compared with the relative proportion of women among new HIV cases according to national report from the Ministry of Health [21]. The general characteristics of HIV-infected women and men were compared using Chi square for categorical values and Mann–Whitney-U tests for continuous values, and the sexual and reproductive health of HIV-infected women was described. The data are presented as line graphs, percentages or median (with interquartile range). Microsoft Excel for Mac 2011 (Microsoft Corporation, Redmond, WA, USA) and Stata version 12 for Mac (Stata Corporation, College Station, TX, USA) were used in the analyses.

## Results

### Trends in women enrolled in care and national epidemiologic trends

**Fig. 1:**
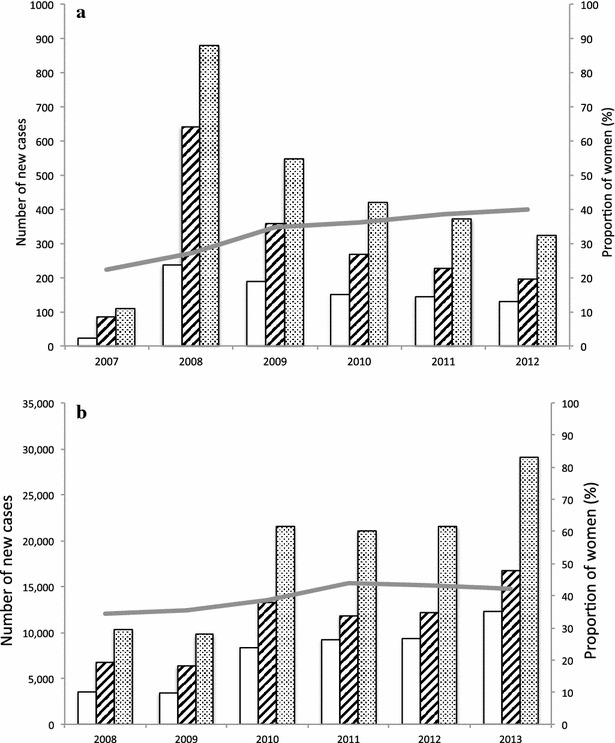
**a** Proportion of female adult patients entering HIV care at Hasan Sadikin Hospital between 2007 and 2012. **b** Proportion of female new HIV cases reported to the Ministry of Health between 2008 and 2013 [21]. *Unfilled bar* Number of women; *striped bar* Number of men; *dotted bar* Total number of new cases; *Grey line* % of women. *Left y-axis* refers to *bar graphs*. *Right y-axis* refers to *line graphs*

Women made up an increasing proportion of the patients enrolled in HIV care in Hasan Sadikin hospital between 2007 and 2012, from 22.2 % (24/108) in 2007 to 39.9 % (129/323) in 2012 (Fig. 1a). The laboratory of this hospital performed HIV tests for 1624 (63.2 %) individuals while the remainder were diagnosed elsewhere; 50.3 % of them in other hospitals or testing facilities in Bandung, 30.6 % in other cities in Indonesia, 1.9 % in correctional facilities, 0.9 % overseas, while for 16.2 % this information was not available. HIV test were repeated for patients who could not provide written HIV test result from other testing facilities. Almost all patients came from the province of West Java and neighbouring Jakarta area—but the gender distribution was in line with national estimates of an increasing proportion of women from 34.4 % in 2008 to 42.3 % in 2013 (Fig. 1b).

### Characteristics of HIV-infected men and women

Women in this cohort were younger, fewer of them had a job and more were divorced or widowed compared to men (Table 1). More women than men reported that their partners have had an HIV test (53.8 vs. 32.6 %, *p* < 0.001) and this was also true for the subgroup of married women and men (73.8 vs 64.6 %, *p* = 0.001). Men and women differed in their probable transmission route (Table 1): significantly fewer women than men (16.1 vs. 73.8 %, *p* < 0.001) reported a history of IDU, and more women had been involved in sex work (6.6 % vs. 0 %, *p* < 0.001) and tested for HIV because of an HIV-positive partner (25.5 vs. 4.0 %, *p* < 0.001)—the two latter numbers were obtained from a subgroup analysis of 212 women and 348 men. Women also presented earlier with less advanced disease, fewer HCV co-infections and a higher CD4 cell count (median: 207 vs. 81 cells/µl; *p* < 0.0001) (Table 1). Among the ART-naïve population, including 499 women and 904 men, CD4 cell counts were much higher in women compared to men (median: 176 vs. 41 cells/µl; *p* < 0.0001). Fewer women than men had received ART prior to enrolment (18.1 vs. 28.4 %, *p* < 0.001). Seventeen women (2.4 %) took ART as PMTCT, two of whom were pregnant when entering care.

**Table 1:** Baseline characteristics of HIV-infected men and women (n = 2662)

		Female* (n = 881)	Male* (n = 1781)
Sociodemographics			
Median age, years (IQR)		28 (25–32)	30 (28–34)
Education, n (%)	No education	2 (0.3)	2 (0.2)
	Up to 6 years	83 (11.3)	44 (2.9)
	>6 to 9 years	110 (14.9)	136 (9.0)
	>9 to 12 years	356 (48.3)	799 (53.1)
	>12 years	186 (25.2)	524 (34.8)
Occupation, n (%)	None	163 (22.1)	340 (22.5)
	Housewife	299 (40.5)	3 (0.2)
	Job in the past month	277 (37.5)	1166 (77.3)
Marital status, n (%)	Single	85 (11.6)	669 (44.5)
	Married	439 (59.7)	709 (47.1)
	Divorced/widowed	211 (28.7)	126 (8.3)
Smoking, n (%)		188 (35.7)	776 (76.0)
Probable transmission route			
History of IDU, n (%)		112 (16.1)	1081 (73.8)
History of sex work, n (%)		14 (6.6)	0
HIV test because of partner notification, n (%)		54 (25.5)	14 (4.0)
Risk behaviour			
Condom use, n (%)	Never	337 (67.8)	427 (56.1)
	Rarely	33 (6.8)	48 (6.4)
	Sometimes	41 (8.3)	66 (8.8)
	Often	14 (2.8)	51 (6.6)
	Always	71 (14.3)	170 (22.1)
Clinical status			
WHO clinical stage, %	I	258 (39.6)	179 (13.5)
	II	58 (8.9)	83 (6.3)
	III	133 (20.4)	399 (30.1)
	IV	203 (31.1)	663 (50.1)
Laboratory parameters			
Median CD4, cells/µl (IQR)		207 (51–370)	81 (18–270)
Positive anti-HCV Antibody, n (%)		122 (22.8)	868 (74.9)
Positive Hepatitis B, n (%)		17 (2.9)	96 (7.7)
Reproductive health			N/A
Pregnant, n (%)		43 (8.6)	
Contraceptive use other than condom, n (%)		120 (22.5)	
Number of children, n (%)	0	179 (25.6)	
	1	320 (45.9)	
	2	142 (20.3)	
	≥3	57 (8.2)	

Data were missing for smoking (40.2 % in women and 42.7 % in men), pregnant (43.1 % in women), condom use (43.6 % in women and 56.8 % in men), history of sex work (75.9 % in women and 80.5 % in men) and HIV test because partner is HIV positive (75.9 % in women and 80.5 % in men)

*IQR* interquartile range, *IDU* injecting drug use, *HCV* hepatitis C virus

* Chi square or Mann–Whitney tests *p* value <0.01 for all variables presented

### Sexual and reproductive health

More than half of the women (56.1 %) never used condoms, less than a quarter of them reported other contraceptive use and some were pregnant at time of enrolment (Table 1). The majority of women had had a steady partner; only 11.6 % were single while the rest were either married or divorced/widowed. Almost all women were in their reproductive age with one quarter of them aged between 16 and 25 years old and less than 1 % older than 49.

The proportion of HIV-infected married women who had disclosed their HIV status to their husbands was higher than disclosure from men to their spouses (75.8 vs. 70.5 %, *p* = 0.057). Likewise, more women indicated that their husbands had been tested for HIV than men indicating their wives had been tested (73.8 vs. 64.6 %, *p* = 0.001).

Forty-three women (8.6 %) were pregnant when entering HIV care (Table 1), but only one was referred to the HIV clinic from an antenatal service. A high proportion of women had at least one child (74.4 %). There were no data of HIV status of these children; but 79 HIV positive children had been enrolled in the cohort with a median age of 3.1 years old and median CD4 cell count of 221 cells/µl.

## Discussion

Our cohort shows an increase in the proportion of women among HIV-infected individuals between 2007 and 2013, in line with national estimates [21]. Compared to men, most women in our cohort were younger and presenting with less advanced disease. Fewer women reported a history of IDU or had HCV coinfection, showing a difference in HIV transmission route. Our findings also indicate that most women were or had been involved in a monogamous relationship: the majority were or had been married, had at least one child, and were not using contraceptives at the time of enrolment. A substantial proportion was pregnant, even though very few were referred from antenatal care.

The growing proportion of women in this cohort can have several explanations. Firstly, the project that started this HIV clinic had focused on IDU as the main transmission route of HIV infection in West Java in 2006 and targeted the PWID accordingly [17, 22]. This project introduced counselling and testing among the female partners of male patient and as more partners were being tested, the number of women entering this cohort increased. Until 2013, HIV screening was not done in women attending antenatal care in Indonesia [14] and HIV testing among sex workers largely relies on individual awareness [23, 24]. Secondly, there might have been a real shift in the route of HIV transmission with IDU becoming less and sexual transmission becoming more important. Even among men, we observed a significant decrease of IDU as HIV transmission risk factor from 80 % in 2008 to 30 % in 2012 in this cohort. National report shows similar estimates: between 2007 and 2011, AIDS cases associated with IDU decreased from 50 to 19 % and cases associated with heterosexual transmission increased from 42 to 71 % [1]. A third factor contributing to a growing proportion of females in this cohort may be an underrepresentation of MSM at the Hasan Sadikin Hospital within the study period. Indeed national projections predict a considerable growth of male HIV patients from 2011 to 2016 due to homosexual transmission [9].

We characterized the risk categories of HIV-infected women in this cohort. In contrast to male patients, few women were PWID based on self-report (16.1 %) and confirmed HCV infection (22.8 %) (Table 1). More than half of the women had a husband who had been HIV tested, but analysis of a subgroup of 212 women only identified 25.5 % as female partners of HIV-infected men diagnosed through partner notification. The subgroup analysis also found 6.6 % women involved in sex work. The number might be an underestimate because the question on sex work was embedded in the following: “In the past month, whom did you have sex with? A) Long-term partner B) Casual partner C) Sex worker D) Client.” In South Africa, 21 % of women attending routine antenatal care was involved in transactional sex associated with HIV seropositivity [25] and in Canada, a surveillance program established the risk categories for female HIV infection as 65 % from heterosexual contact and 25 % from IDU [26]. Clearly, sex work and other routes of HIV transmission need further study among HIV-infected women in Indonesia.

Almost all women in this cohort were in their reproductive age and sexually active. Compared to women of reproductive age with unknown HIV status in the 2012 general demographic and health survey (DHS), more women in this cohort were divorced or widowed (28.7 vs. 4.9 %) [27]. The high mortality among HIV-infected men in this cohort [28] might contribute to the high number of widowed women. A very high divorce rate among FSW (74 %) [29] suggests that the relatively high proportion of widowed or divorced women in our cohort may also be due to undetected FSW. Few women remain without a partner for a long time in the aftermath of a husband’s death, they remarry to protect their children’s interests [30] and avoid the public stigma of being a divorcee or a widow [31]. A woman may not disclose her HIV status to an HIV-negative new partner [32], thus exposing him to a risk of transmission without proper prevention strategies.

Compared to men, women had less advanced disease, with higher CD4 cell counts and fewer complications, probably because women were mostly tested because of their husband’s illness (Table 1). Testing of sexual partners of PWID is important in this setting; a cross-sectional study in a similar setting showed that this particular subgroup have a very high HIV risk [33]. Screening of women in antenatal care is not routinely done in Indonesia; indeed only one out of the 43 pregnant women in this cohort was referred from an obstetric care. This is in contrast to sub-Saharan African settings where many women are diagnosed in antenatal screening as part of the prevention of mother-to-child transmission (PMTCT) program [34]. The Indonesian Ministry of Health Program Monitoring in Universal Access documented only 0.4 % (21,103) out of 5,060,637 estimated pregnancies in Indonesia in 2011 tested for HIV [1]—and these tests were likely to be prompted by other risk characteristics identified instead of a screening in general population.

Our findings also raise the issue of family planning for HIV-infected women in Indonesia. Women in this cohort differ from women in general population surveyed in the DHS [27]: more were pregnant (8.6 vs. 4.3 %) and fewer use contraceptives (22.5 vs. 44.4 %) at the time of the cross-sectional data collection. This may imply that more women in this cohort were in a monogamous relationship at the time of enrolment, although it also highlights the need to focus on preconception and contraceptive care for HIV-infected women [35]. Younger women with no or one child generally desire more children [36] and in Indonesia, the social status associated with fertility [14] and religious values attributed to having children [37] may also influence childbearing desire. A lack of knowledge on mother-to-child transmission (MTCT) among HIV-infected women [38] increases the potential for vertical HIV transmission, but data on HIV in children to confirm this case is scarce. Indonesia adopted PMTCT as a national policy in 2005 [14] but by 2011 only 7.38 % of women in need of PMTCT are getting the service [9] mainly in the form of prophylactic ARV, formula milk support and counselling.

This study was conducted in a single referral hospital, albeit serving around 35 % of HIV-infected individuals in West Java [21]. Because it was not obtained from routine screening, the number of HIV infection in this cohort may not represent a true prevalence of HIV in the general female population hence data should be interpreted with caution. Commercial sex workers and MSM as key populations with high prevalence rates [39] may be underrepresented, limiting the generalizability of our findings. Moreover, some of the information relied on self-report, which may be subject to a variety of biases including social desirability bias [40]. More than 40 % of women have missing data on pregnancy status but there were no significant differences in age, education level, home address or marital status between those with missing or non-missing pregnancy status [41]. Finally, no information was available to identify couples of HIV-infected men and women, which might help establish transmission routes. The information gaps highlighted by this study may be used to guide further research.

## Conclusion

The number of HIV-infected women in Indonesia is increasing and they might be bridging the HIV epidemic to a wider population in relation to their sexual and reproductive health. Further research to elucidate the characteristics of women exposed to HIV, their access to testing and care and sexual and reproductive needs can help prevent transmission to women and children in the context of the concentrated HIV epidemic in Indonesia.

## Abbreviations

AEM: Asian Epidemic Model
AIDS: acquired immune deficiency syndrome
ART: antiretroviral therapy
cART: combined antiretroviral therapy
DHS: demographic and health survey
FSW: female sex workers
HBV: Hepatitis B virus
HCV: hepatitis C virus
HIV: human immunodeficiency virus
HIV-RNA: human immunodeficiency virus-ribonucleic acid
IDU: injecting drug use
MSM: men who have sex with men
MTCT: mother-to-child transmission of HIV
PITC: provider-initiated testing and counselling
PMTCT: prevention of mother-to-child transmission of HIV
PWID: people who inject drugs
UNAIDS: Joint United Nations Programme on HIV/AIDS
VCT: voluntary counselling and testing

## References

[1] Indonesian National AIDS Commission. Republic of Indonesia Country Report on the Follow up to the Declaration of Commitment on HIV/AIDS (UNGASS) Reporting Period 2010–2011. 2012.

[2] Directorate General CDC and EH Ministry of Health (2011). Integrated biological and behavioural survey 2011.

[3] UNAIDS. HIV in Asia and the Pacific [Internet]. 2013. http://www.unaids.org/sites/default/files/media_asset/2013_HIV-Asia-Pacific_en_0.pdf.

[4] Indonesian General Directorate of Communicable Disease Control and Environmental Health. [Cases of HIV/AIDS in Indonesia reported through September 2014] Statistik Kasus HIV/AIDS di Indonesia Dilapor s/d September 2014. Jakarta. 2014.

[5] Pisani E, Dadun, Sucahya PK, Kamil O, Jazan S. Sexual behavior among injection drug users in 3 indonesian cities carries a high potential for HIV spread to noninjectors. J Acquir Immune Defic Syndr [Internet]. 2003;34(4):403–6. http://www.ncbi.nlm.nih.gov/pubmed/14615658. 10.1097/00126334-200312010-00007 14615658

[6] Pisani E, Girault P, Gultom M, Sukartini N, Kumalawati J, Jazan S, et al. HIV, syphilis infection, and sexual practices among transgenders, male sex workers, and other men who have sex with men in Jakarta, Indonesia. Sex Transm Infect [Internet]. 2004;80(6):536–40. http://www.pubmedcentral.nih.gov/articlerender.fcgi?artid=1744942&tool=pmcentrez&rendertype=abstract. 10.1136/sti.2003.007500 PMC1744942 15572631

[7] Morineau G, Bollen LJ, Syafitri RI, Nurjannah N, Mustikawati DE, Magnani R. HIV prevalence and risk behaviours among injecting drug users in six indonesian cities implications for future HIV prevention programs. Harm Reduct J [Internet]. 2012;9(1):37. http://www.pubmedcentral.nih.gov/articlerender.fcgi?artid=3494521&tool=pmcentrez&rendertype=abstract. 10.1186/1477-7517-9-37 PMC3494521 22943438

[8] Des Jarlais DC, Feelemyer JP, Modi SN, Arasteh K, Mathers BM, Degenhardt L, et al. Transitions from injection-drug-use-concentrated to self-sustaining heterosexual HIV epidemics: patterns in the international data. PLoS One [Internet]. 2012;7(3):e31227. http://www.pubmedcentral.nih.gov/articlerender.fcgi?artid=3291614&tool=pmcentrez&rendertype=abstract. 10.1371/journal.pone.0031227 PMC3291614 22396729

[9] Republic of Indonesia Ministry of Health. Estimates and Projection of HIV/AIDS in Indonesia 2011–2016 [Internet]. Jakarta. 2014. http://www.ino.searo.who.int/LinkFiles/HIV-AIDS_and_sexually_transmitted_infections_Estimates_and_Projection_HIV_AIDS_ENGLISH.pdf.

[10] Iskandar S, Basar D, Hidayat T, Siregar IM, Pinxten L, Van Crevel R, et al. High risk behavior for HIV transmission among former injecting drug users: a survey from Indonesia. BMC Public Health [Internet]. BioMed Central. 2010;10(1):472. http://www.pubmedcentral.nih.gov/articlerender.fcgi?artid=2931466&tool=pmcentrez&rendertype=abstract. 10.1186/1471-2458-10-472 PMC2931466 20698979

[11] Delvaux T, Nöstlinger C. Reproductive choice for women and men living with HIV: contraception, abortion and fertility. Reprod Health Matters [Internet]. 2007; 15(29 Suppl):46–66. http://www.ncbi.nlm.nih.gov/pubmed/17531748. 10.1016/S0968-8080(07)29031-7 17531748

[12] Anderson JR, Cohan D, Cu-Uvin S. HIV and reproduction: fertility, contraception, and preconception issues and interventions. Infect Dis Obstet Gynecol [Internet]. 2012;2012:736864. http://www.pubmedcentral.nih.gov/articlerender.fcgi?artid=3546473&tool=pmcentrez&rendertype=abstract. 10.1155/2012/736864 PMC3546473 23345960

[13] Kendall T, Bärnighausen T, Fawzi WW, Langer A (2014). Towards Comprehensive Women’ s Healthcare in Sub-Saharan Africa : addressing intersections between HIV, reproductive and maternal health. J Acquir Immune Defic Syndr.

[14] Hardon AP, Oosterhoff P, Imelda JD, Anh NT, Hidayana I. Preventing mother-to-child transmission of HIV in Vietnam and Indonesia: diverging care dynamics. Soc Sci Med [Internet]. 2009;69(6):838–45. http://www.ncbi.nlm.nih.gov/pubmed/19576671. 10.1016/j.socscimed.2009.05.043 19576671

[15] Surya IGP, Kornia K, Suwardewa TGA, Mulyanto, Tsuda F, Mishiro S. Serological markers of hepatitis B, C, and E viruses and human immunodeficiency virus type-1 infections in pregnant women in Bali, Indonesia. J Med Virol [Internet]. 2005;75(4):499–503. http://www.ncbi.nlm.nih.gov/pubmed/15714491. 10.1002/jmv.20314 15714491

[16] Muhaimin T, Besral. HIV Prevalence among Pregnant Women in Eight Provincial Capitals in Indonesia in 2003–2010. PREVALENSI HIV PADA IBU HAMIL DI DELAPAN IBU KOTA PROVINSI DI INDONESIA TAHUN 2003–2010. Makara Kesehat. 2011;15(2):93–100.

[17] Pinxten WJL, Ia T, Hospers HJ, Alisjahbana B, Meheus AM, Van RC, et al. IMPACT-Bandung: a learning organization approach to build HIV prevention and care in Indonesia. Procedia Soc Behav Sci [Internet]. 2011;15(0):623–7. http://www.sciencedirect.com/science/article/pii/S1877042811003314.

[18] Statistics Indonesia (Badan Pusat Statistik-BPS). Population Indonesia by Province [Internet]. 2012. pp. 1. http://www.bps.go.id/eng/tab_sub/view.php?tabel=1&id_subyek=12.

[19] Wisaksana R, Alisjahbana B, van Crevel R, Kesumah N, Sudjana P, Sumantri R. Challenges in delivering HIV-care in Indonesia: experience from a referral hospital. Acta Med Indones [Internet]. 2009;41(Suppl 1):45–51. http://www.ncbi.nlm.nih.gov/pubmed/19920298. 19920298

[20] Nelwan EJ, Van Crevel R, Alisjahbana B, Indrati AK, Dwiyana RF (2010). Human immunodeficiency virus, hepatitis B and hepatitis C in an Indonesian prison: prevalence, risk factors and implications of HIV screening. Trop Med Int Health..

[21] Directorate General CDC and EH Ministry of Health. HIV and AIDS Situational Report in Indonesia 2013. Laporan Situasi Perkembangan HIV and AIDS di Indonesia Tahun 2013. Jakarta. 2013.

[22] Afriandi I, Aditama TY, Mustikawati D, Oktavia M, Alisjahbana B, Riono P. HIV and injecting drug use in Indonesia: epidemiology and national response. Acta Med Indones [Internet]. 2009;41(Suppl 1):75–8. http://www.ncbi.nlm.nih.gov/pubmed/19920303. 19920303

[23] Kurniawati L, Lina M, Kumalasari F, Wulandari R. Analysis of factors impeding the utilization of HIV voluntary counseling and testing (VCT) among commercial sex workers in Surakarta. Analisis Hambatan Pemanfaatan Voluntary Counseling and Testing (VCT) Pada Pekerja Seks Komersial di Surakarta Dalam Ran. J KesMaDaSka. 2014;(104):35–41.

[24] Shannon K, Goldenberg SM, Deering KN, Strathdee SA. HIV infection among female sex workers in concentrated and high prevalence epidemics: why a structural determinants framework is needed. Curr Opin HIV AIDS [Internet]. 2014;9(2):174–82. http://www.ncbi.nlm.nih.gov/pubmed/24464089. 10.1097/COH.0000000000000042 PMC4286412 24464089

[25] Dunkle KL, Jewkes RK, Brown HC, Gray GE, McIntryre JA, Harlow SD (2004). Transactional sex among women in Soweto, South Africa: prevalence, risk factors and association with HIV infection. Soc Sci Med..

[26] Forbes JC, Alimenti AM, Singer J, Brophy JC, Bitnun A, Samson LM, et al. A national review of vertical HIV transmission. AIDS [Internet]. 2012;26(6):757–63. http://www.ncbi.nlm.nih.gov/pubmed/22210635. 10.1097/QAD.0b013e328350995c 22210635

[27] Statistics Indonesia (Badan Pusat Statistik-BPS), National Population and Family Planning Board (BKKBN), Republic of Indonesia Ministry of Health. Indonesia demographic and health survey 2012. Jakarta. 2013.

[28] Wisaksana R, Indrati AK, Fibriani A, Rogayah E, Sudjana P, Djajakusumah TS, et al. Response to first-line antiretroviral treatment among human immunodeficiency virus-infected patients with and without a history of injecting drug use in Indonesia. Addiction [Internet]. 2010;105(6):1055–61. http://www.ncbi.nlm.nih.gov/pubmed/20331555. 10.1111/j.1360-0443.2010.02898.x 20331555

[29] Kementerian Kesehatan Republik Indonesia. Integrated Biological and Behavioral Survey 2007. Laporan Survei Terpadu Biologi dan Perilaku tahun 2007. Jakarta. 2009.

[30] Damar AP, du Plessis G. Coping versus grieving in a “Death-accepting” society: AIDS-bereaved women living with HIV in Indonesia. J Asian Afr Stud [Internet]. 2010;45(4):424–31. http://jas.sagepub.com/cgi/doi/10.1177/0021909610373904. 10.1177/0021909610373904 20827839

[31] Irianti M. Community Perception on divorced or widowed women [Persepsi masyarakat terhadap status janda]. Jember. 2007.

[32] Anindita M, Shaluhiyah Z, Suryoputro A. Non disclosure of HIV positive status of women to their partner : implication for PMTCT in Central Java Indonesia. Sci J Med Clin Trials. 2013;1–6.

[33] Hammett TM, Phan S, Nguyen P, Kieu B, Dang S, Nguyen D (2015). Female sexual partners of male people who inject drugs in vietnam have poor knowledge of their male partners’ HIV status..

[34] Hensen B, Baggaley R, Wong VJ, Grabbe KL, Shaffer N, Lo Y-RJ, et al. Universal voluntary HIV testing in antenatal care settings: a review of the contribution of provider-initiated testing & counselling. Trop Med Int Health [Internet]. 2012;17(1):59–70. http://www.ncbi.nlm.nih.gov/pubmed/22032300. 10.1111/j.1365-3156.2011.02893.x 22032300

[35] Hoyt MJ, Storm DS, Aaron E, Anderson J. Preconception and contraceptive care for women living with HIV. Infect Dis Obstet Gynecol [Internet]. 2012:604183. http://www.pubmedcentral.nih.gov/articlerender.fcgi?artid=3477542&tool=pmcentrez&rendertype=abstract. 10.1155/2012/604183 PMC3477542 23097595

[36] Withers M, Kano M, Pinatih GNI (2010). Desire for more children, contraceptive use and unmet need for family planning in a remote area of Bali, Indonesia. J Biosoc Sci..

[37] Instone S, Mueller M-R. Religious influences on the reproductive health decisions of HIV-positive Latinas on the border. J Relig Health [Internet]. 2011;50(4):942–9. http://www.ncbi.nlm.nih.gov/pubmed/19937387. 10.1007/s10943-009-9307-1 19937387

[38] Oktavia M, Alban A, Zwanikken PA. A qualitative study on HIV positive women experience in PMTCT program in Indonesia. Retrovirology [Internet]. BioMed Central Ltd; 2012;9(Suppl 1):P119. http://www.retrovirology.com/content/9/S1/P119.

[39] Dokubo EK, Kim AA, Le LV, Nadol PJ, Prybylski D, Wolfe MI. HIV incidence in Asia: a review of available data and assessment of the epidemic. AIDS Rev [Internet]. University of California San Francisco, Center for AIDS Prevention Studies, San Francisco, CA, USA. KDokubo@cdc.gov; 2013;15(2):67–76. http://europepmc.org/abstract/MED/23681434. 23681434

[40] Kelly CA, Soler-Hampejsek E, Mensch BS, Hewett PC. Social desirability bias in sexual behavior reporting: Evidence from an interview mode experiment in rural Malawi. International Perspectives on Sexual and Reproductive Health. 2013. pp. 14–21. 10.1363/3901413 PMC4023461 23584464

[41] Sterne JAC, White IR, Carlin JB, Spratt M, Royston P, Kenward MG (2009). Multiple imputation for missing data in epidemiological and clinical research: potential and pitfalls. Br Med J.

